# Case report: A pregnant woman accidental treated with spironolactone in mid-gestation

**DOI:** 10.3389/fphar.2024.1404251

**Published:** 2024-07-25

**Authors:** Nianying Deng, Jiayi Zhong, Zhengjun Deng, Minling Chen, Liangqi Yan, Haiting Li, Jiawei Han, Enfu Tao

**Affiliations:** ^1^ Department of Pharmacy, Wenling Maternal and Child Health Care Hospital, Wenling, Zhejiang, China; ^2^ Department of Maternity, Wenling Maternal and Child Health Care Hospital, Wenling, Zhejiang, China; ^3^ Department of Ultrasound, Wenling Maternal and Child Health Care Hospital, Wenling, Zhejiang, China; ^4^ Department of Neonatology and NICU, Wenling Maternal and Child Health Care Hospital, Wenling, Zhejiang, China; ^5^ Department of Science and Education, Wenling Maternal and Child Health Care Hospital, Wenling, Zhejiang, China

**Keywords:** spironolactone, pregnancy, feminization, antiandrogenic effects, fetal safety

## Abstract

Spironolactone, a potassium-sparing diuretic, is used to treat hypertension, heart failure, and certain hyperandrogenic disorders. Its use during pregnancy is not recommended due to the risk of feminizing male fetuses, primarily because of its antiandrogenic activity. However, human data remain scarce and largely inconclusive. Here, we present the first case of a 25-year-old pregnant woman, at 16 weeks of gestation, who was inadvertently exposed to spironolactone (240 mg/day) for 1 week due to a pharmacy dispensing error. The patient subsequently delivered a healthy male infant with normal genitalia at 38 weeks of gestation following vaginal delivery. Current follow-up shows that the infant is healthy and developing normally. This article summarizes the potential causes of spironolactone-induced anomalous genital development and explores the safety of new-generation mineralocorticoid receptor antagonists (MRAs) during pregnancy. The mechanisms behind spironolactone-induced anomalous genital development in male fetuses have not been fully elucidated. Spironolactone competes with dihydrotestosterone for binding to androgen receptors and inhibits enzymes involved in androgen biosynthesis, which may partly explain its antiandrogenic effects. Recent advancements in MRAs have led to the development of compounds with higher selectivity for the mineralocorticoid receptor, thereby reducing the incidence of antiandrogen side effects. These new-generation MRAs may be effective alternatives during pregnancy, but more data are needed to establish their safety in pregnant women. This case contributes to the limited but growing body of literature on the safety profile of spironolactone in pregnancy, providing insights into its effects during a critical period of fetal development.

## Introduction

Spironolactone, a steroid that functions as both a potassium-sparing diuretic and an antiandrogen, has been widely used to manage various conditions, including hypertension (Tian et al., 2024), heart failure (Pantelidis et al., 2018), and certain hyperandrogenic disorders (Carmina et al., 2022; Sabbadin et al., 2023). Its use during pregnancy is not recommended due to potential teratogenic effects, primarily stemming from its antiandrogenic activity. Animal studies have indicated that exposure to spironolactone during critical periods of fetal development can lead to the feminization of male offspring (Hecker et al., 1980; Liszewski and Boull, 2019). However, human data remain scarce and largely inconclusive.

Against this backdrop, we present a case of mid-gestation exposure to spironolactone resulting from a pharmacy dispensing error. This case highlights the issue of pharmacy dispensing errors, particularly within the vulnerable population of pregnant women. Our case study aims to provide valuable reference data on spironolactone exposure during pregnancy, aiding patients and healthcare providers in making informed decisions while navigating the delicate balance of benefits and risks.

### Case presentation

A 25-year-old woman, previously in good health with no history of drug use, family history, infectious disease, or genetic disease, presented at 16 1/7 weeks of gestation with abnormal liver function tests (LFTs) detected at an external hospital: alanine aminotransferase (ALT) 209 IU/L and aspartate aminotransferase (AST) 85 IU/L. She had irregular menstruation and was gravida 1, para 0 (G1P0). Upon discharge, she was prescribed glucuronolactone tablets (200 mg) and vitamin C tablets (200 mg), each to be taken three times daily (tid). During a follow-up visit at 17 1/7 weeks of gestation, her LFTs showed further escalation (ALT 237 IU/L, AST 76 IU/L). The prescribed regimen of glucuronolactone 200 mg tid was continued. However, upon returning home, she noticed a discrepancy in the medication packaging and discovered that the tablets dispensed at 16 1/7 weeks were not glucuronolactone but spironolactone 80 mg tid (240 mg/d). By then, she had inadvertently consumed a total of 84 spironolactone tablets over 1 week.

**FIGURE 1:**
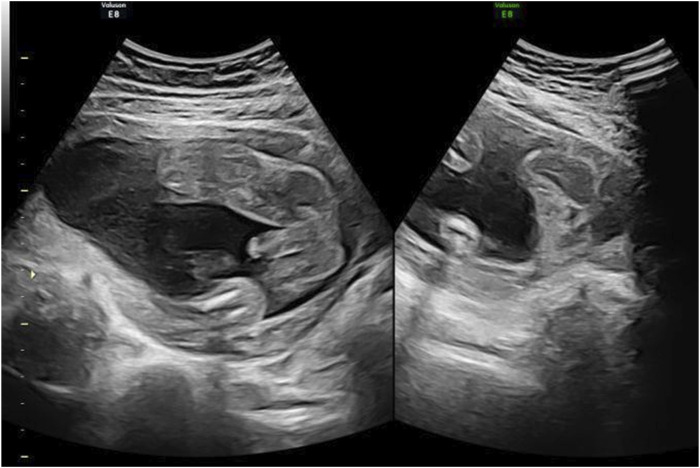
4D ultrasound of genital development at 24 weeks in the spironolactone-exposed pregnancy. The ultrasound shows no obvious abnormalities in the fetus.

**FIGURE 2:**
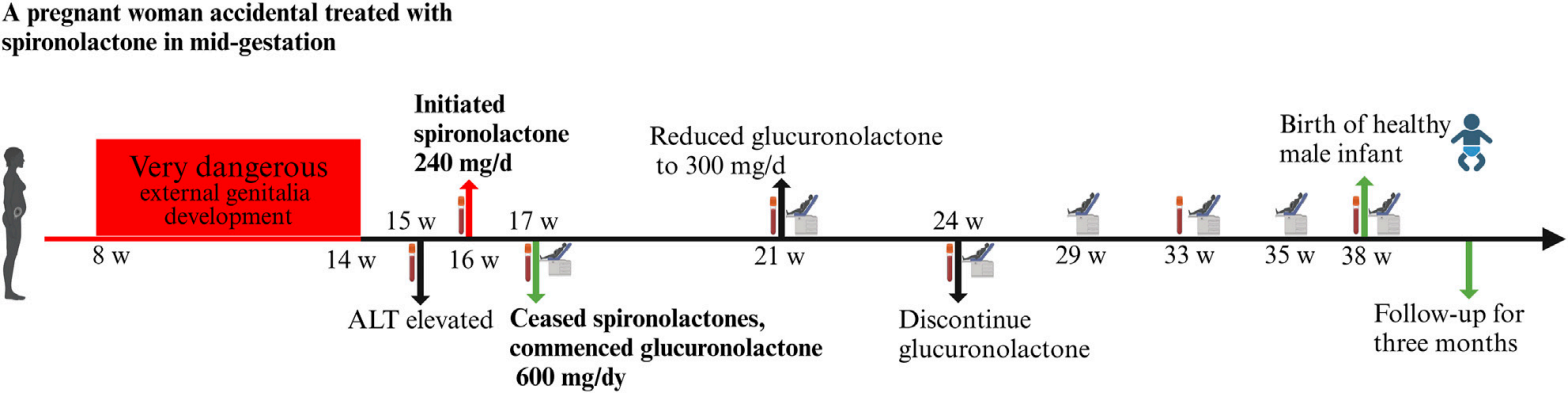
The entire treatment and monitoring process from spironolactone exposure to postnatal follow-up in the spironolactone-exposed pregnancy. Following spironolactone exposure, the prenatal testing plan includes hepatitis screening (week 16), Down’s syndrome screening (week 17), and non-invasive DNA testing with a biochemical exam (week 21). Routine blood tests and biochemical assessments are planned for weeks 24 and 33. A thorough evaluation at week 38 includes routine blood tests, biochemical analysis, coagulation checks, blood grouping, irregular antibody screening, and preoperative immunization.

Subsequent obstetric examination and fetal ultrasound assessment revealed normal development of the fetal genitalia (Figure 1). After being informed of the risks associated with inadvertent spironolactone exposure, particularly the potential for feminization of a male fetus, the patient chose to continue with the pregnancy. As her pregnancy progressed, her LFTs improved, with ALT and AST levels decreasing to 155 IU/L and 47 IU/L respectively by 21 4/7 weeks of gestation and returning to the normal range by 24 1/7 weeks of gestation. During this period, routine obstetric examinations, fetal ultrasound assessments, Down’s syndrome screening, and non-invasive DNA prenatal screening were performed and were normal. Consequently, glucuronolactone tablets were tapered down to 100 mg tid and eventually discontinued. At 38 1/7 weeks of gestation, the patient was admitted to the hospital with spontaneous rupture of membranes and loss of amniotic fluid. The patient delivered a male infant weighing 3,400 g, with an Apgar score of 10/10 at 1 and 5 min. The delivery was uncomplicated, with no obstetric issues. A highly experienced neonatologist conducted a thorough physical examination of the newborn. The neonate was alert and active with a body temperature of 36.5°C, a heart rate of 140 beats per minute, a respiratory rate of 50 breaths per minute, a length of 50.5 cm, and a head circumference of 34 cm, with no rashes or petechiae. Breath sounds were clear bilaterally with no rales, and the heart rate was regular with no murmurs. The abdomen was flat and soft, without hepatosplenomegaly. The limbs were active with normal primitive reflexes. The external genitalia were normal with bilaterally descended testes and well-formed scrotal folds, with no signs of hypospadias or other malformations. Complete blood count and blood biochemistry results showed no significant abnormalities. Ultrasound examinations of the head, heart, abdomen, urinary system, and scrotum showed no abnormalities. Unfortunately, the placenta tissue was not collected and evaluated due to the request of the patient’s family. Follow-up interviews were conducted until 3 months after delivery. Based on the medical examination results, the infant showed no abnormalities from birth, including both genital appearance and blood parameters. The mother remained in good health and continued to breastfeed consistently. The progression of laboratory parameters following spironolactone administration is detailed in Table 1. The entire treatment and monitoring process from spironolactone exposure to postnatal follow-up in the spironolactone-exposed pregnancy is outlined in Figure 2.

**TABLE 1:** Time course of laboratory parameters in relation to the administration of spironolactone.

Gestation period (week)	Days after stopping spironolactone	ALT (IU/L)	AST (IU/L)	Event
16 1/7	0	209	85	Initiated spironolactone 240 mg/day
17 1/7	0	237	76	Error identified
17 2/7	1	224	80	Ceased spironolactone, commenced glucuronolactone 600 mg/d
21 4/7	24	155	47	Reduced glucuronolactone to 300 mg/d
24 1/7	42	24	17	Discontinued glucuronolactone
38 1/7	243	6	10	Birth of healthy male infant

ALT, alanine aminotransferase; AST, aspartate aminotransferase.

## Discussion

To our knowledge, the case of a pregnant woman accidentally treated with spironolactone due to pharmacy dispensing errors has not been previously reported. There has been insufficient study on the rate of pharmacy dispensing errors. However, a recent systematic review and meta-analysis quantified the worldwide prevalence of dispensing errors across communities, hospitals, and other pharmacy settings in literature published between 2010 and 2023. The prevalence of dispensing errors ranged from 0% to 33.3%, and the pooled prevalence for dispensing errors was 1.6% overall (Um et al., 2024). Beyond that, the incidence of pharmacy dispensing errors in the pregnant population is unknown. The consequences of medication errors can be severe, especially in pregnant women. As a result of this pharmacy error, the pregnant woman developed an adverse drug reaction such as oligohydramnios (Grincevičienė et al., 2016), abdominal pain, constipation (Einarson et al., 1999), gallbladder disease (Mertl and Kelly, 1999), and even spontaneous abortion (Einarson et al., 1999). Concern about dispensing errors in pregnancy must focus not only on the pregnant woman but also on the fetus, who is placed at potential risk for a wide range of adverse effects. These errors serve to remind pharmacists that they need to take great care when dispensing drugs, especially for pregnant women. Fortunately, in our case, the mother experienced no pregnancy complications, and the male infant was healthy and exhibited no signs of feminization.

Spironolactone, a potassium-sparing diuretic, operates by competitively binding to aldosterone receptor sites in the distal renal tubules, facilitating increased sodium chloride and water excretion and the conservation of potassium and hydrogen ions. Its broad spectrum of clinical applications includes the treatment of primary aldosteronism (Forestiero et al., 2023), hypertension (Tian et al., 2024), heart failure (Kjeldsen et al., 2020), and various androgen-mediated skin disorders such as acne, hirsutism, female-pattern baldness (Bienenfeld et al., 2019; Burns et al., 2020), androgenetic alopecia (James et al., 2022), and Bartter syndrome (Konrad et al., 2021). Its use during pregnancy is controversial due to reports of teratogenic effects in animal models (Hecker et al., 1980; Jaussan et al., 1985). A systematic review reported that feminization of exposed males was mentioned in six of nine animal studies, and five studies used more than the human equivalent doses of 200 mg/day (Liszewski and Boull, 2019). However, human data remain scarce and largely inconclusive. To our knowledge, only two cases of males born with ambiguous genitalia following maternal exposure to spironolactone have been documented (Shah, 2011; Levy et al., 2023). The first case reported at the 2011 Endocrinology Annual Meeting describes an instance where a male newborn presented with feminization attributed to the mother’s use of spironolactone from the beginning of pregnancy up to the fifth week of gestation (Shah, 2011). The other recent case report noted genital anomalies in a newborn linked to maternal exposure to spironolactone and dutasteride until 8 weeks of gestation (Levy et al., 2023). By contrast, several cases in which mothers were treated with spironolactone during their pregnancies reported no adverse effects on the newborns (Groves and Corenblum, 1995; de Arriba et al., 2009; Pieper, 2015). Notably, a woman with Bartter’s syndrome treated with up to 400 mg/day of spironolactone through her first and second trimesters delivered two healthy male infants and one healthy female infant. Both boys exhibited no signs of feminization, and the oldest child was followed up to age thirteen. Both boys appear to have a mild learning disability but have otherwise progressed well (Groves and Corenblum, 1995). This case might indicate that prenatal spironolactone exposure could have cognitive or behavioral effects on children, but further research is required. Unfortunately, based on the available information, it is not clear what potential long-term effects or developmental delays in the fetus may result from pregnancy spironolactone exposure. Our current follow-up outcome shows that this infant is healthy and developing normally. Follow-up should continue to assess long-term outcomes according to the maternal and child health system of the city. In addition, further research is needed to better understand the risks and long-term implications of spironolactone exposure during pregnancy.

External genitalia development is complete at 14 weeks of gestation (Jost, 1966). During embryogenesis, testosterone (T) and dihydrotestosterone (DHT) are the two main androgens that determine the embryologic development of the male reproductive organs and genitalia (Pinson et al., 2023). In the presence of the enzyme 5alpha-reductase, T is converted to the more potent DHT, which has a greater binding affinity for androgen receptors (AR) (Murashima et al., 2015). Complex signaling pathways via AR signal the development of sexual organs and genitalia from both the epithelium and mesenchymal fetal structures (Levy et al., 2023). The mechanism by which spironolactone causes estrogenic effects is not fully understood. One convincing explanation is that spironolactone competes with DHT for the intracellular AR sites *in vivo* (Bonne and Raynaud, 1974; Corvol et al., 1975). Furthermore, spironolactone acts as an antiandrogen in the liver, decreasing both AR and male-specific estrogen binder levels in male rate experiments, resulting in a significant decrease in plasma T levels (Francavilla et al., 1987). Another possible mechanism is that spironolactone reduces the activity of 17-hydroxylase by decreasing microsomal cytochrome P450 in the adrenal and testis, which affects T synthesis (Menard et al., 1974). Additionally, an alteration of testosterone-estrogen balance may partly explain the antiandrogenic actions of spironolactone (Chopra et al., 1973). The reduction of androgens during pregnancy may have detrimental effects on the development of the fetus’s external genitalia. Fortunately, this patient was exposed to spironolactone after 14 weeks of gestation, and the genitalia of the fetus were normal. Our case indicates that the antiandrogenic effects of spironolactone may primarily impact the critical developmental period of external genitalia. After this period, the impact may be less significant. However, more research is needed to support our findings.

Due to the adverse effects of spironolactone, there has been a drive to find more selective mineralocorticoid receptor antagonists (MRAs) with fewer adverse effects. The second generation of steroidal MRAs, such as eplerenone (FDA pregnancy category B) (Craft, 2004), may be more specific to the mineralocorticoid receptor (MR), reducing off-target effects (Agarwal et al., 2021) and appears to be a safe and effective alternative in managing primary aldosteronism during pregnancy (Riester and Reincke, 2015). Compared with spironolactone, treatment with eplerenone is associated with a lower rate of hormonal side effects due to its increased MR specificity (Struthers et al., 2008). Recent research has focused on nonsteroidal MRAs, which are highly selective for MR without antagonistic actions on glucocorticoid, androgen, or progesterone receptors (Pandey et al., 2022). Esaxerenone and finerenone are the only two approved for treatment globally in various regions (Kolkhof et al., 2021). Recently, a case was reported of a pregnant woman treated safely with esaxerenone, despite her advanced maternal age and diagnosis of idiopathic hyperaldosteronism (IHA) and superimposed preeclampsia (SPE) (Yamashita et al., 2022). There are no available data on the use of finerenone in pregnancy to evaluate the drug-associated risk of major birth defects, miscarriage, or adverse maternal or fetal outcomes (Dey et al., 2024). Fetal toxicity has been observed with the use of this drug in animal models (Finerenone Kerendia for chronic kidney disease, 2021). Further research is needed to determine the safety of MRAs use during pregnancy.

Our case study comes with several noteworthy limitations. Firstly, it is merely a single case report, which limits the breadth of its implications. Secondly, the unintended use of spironolactone in a pregnant woman experiencing liver function abnormalities during mid-pregnancy has reignited debates concerning the medication’s safety during gestation. Lastly, although maternal androgen transfer to the fetus occurs through the placenta, our study did not assess hormone levels in the placenta and amniotic fluid, thereby missing potentially crucial data on endocrine interactions.

## Conclusion

This case study presents a 25-year-old pregnant woman at 16 weeks of gestation who was inadvertently exposed to spironolactone (240 mg/day) for 1 week due to a pharmacy dispensing error. The patient delivered a healthy male infant with normal genitalia at 38 weeks of gestation following vaginal delivery. Spironolactone competes with dihydrotestosterone for binding to androgen receptors and inhibits enzymes involved in androgen biosynthesis, which may partly explain its antiandrogenic actions. Compared to spironolactone, eplerenone has increased MR specificity and appears to be a safe and effective alternative during pregnancy. Esaxerenone and finerenone are the only two approved non-steroidal MRAs. However, more data are needed to confirm the safety of these drugs in pregnant women. This single case shows that pregnant women exposed to spironolactone in early and mid-gestation may not be at as high a risk as previously thought on animal research. However, this does not mean that monitoring for potential adverse outcomes should be reduced throughout pregnancy. This case contributes to the limited but growing body of literature on the safety profile of spironolactone in pregnancy, providing insights into the drug’s effects during a critical period of fetal development.

## Data Availability

The original contributions presented in the study are included in the article/Supplementary Material, further inquiries can be directed to the corresponding author.
